# Decoupling environmental effects and host population dynamics for anthrax, a classic reservoir-driven disease

**DOI:** 10.1371/journal.pone.0208621

**Published:** 2018-12-12

**Authors:** Juan Pablo Gomez, Dawn M. Nekorchuk, Liang Mao, Sadie J. Ryan, José Miguel Ponciano, Jason K. Blackburn

**Affiliations:** 1 Spatial Epidemiology and Ecology Research Laboratory, Department of Geography, University of Florida, Gainesville, Florida, United States of America; 2 Emerging Pathogens Institute, University of Florida, Gainesville, Florida, United States of America; 3 Department of Biology, University of Florida, Gainesville, Florida, United States of America; 4 Geospatial Sciences Center of Excellence, South Dakota State University, Brookings, South Dakota, United States of America; 5 Quantitative Disease Ecology and Conservation Lab, Department of Geography, University of Florida, Gainesville, Florida, United States of America; Universite des Montagnes, CAMEROON

## Abstract

Quantitative models describing environmentally-mediated disease transmission rarely focus on the independent contribution of recruitment and the environment on the force of infection driving outbreaks. In this study we attempt to investigate the interaction between external factors and host’s population dynamics in determining the outbreaks of some indirectly transmitted diseases. We first built deterministic and stochastic compartmental models based on anthrax which were parameterized using information from literature and complemented with field observations. Our force of infection function was derived modeling the number of successful transmission encounters as a pure birth process that depends on the pathogen’s dispersion effort. After accounting for individual heterogeneity in pathogen’s dispersion effort, we allowed the force of infection to vary seasonally according to external factors recreating a scenario in which disease transmission increases in response to an environmental variable. Using simulations we demonstrate that anthrax disease dynamics in mid-latitude grasslands is decoupled from hosts population dynamics. When seasonal forcing was ignored, outbreaks matched hosts reproductive events, a scenario that is not realistic in nature. Instead, when allowing the force of infection to vary seasonally, outbreaks were only present in years were environmental variables were appropriate for the outbreaks to develop. We used the stochastic formulation of the force of infection to derive *R*_0_ under scenarios with different assumptions. The derivation of *R*_0_ allowed us to conclude that during epizootic years, pathogen contribution to disease persistence is nearly independent of dispersion. In endemic years, only pathogens with high dispersion significantly prevent disease extinction. Finally, we used our model in a maximum likelihood framework to estimate the parameters that determined a significant anthrax outbreak in Montana in 2008. Our study highlights the importance of the environment in determining anthrax outbreak intensity and could be useful to predict future events that could result in significant wildlife and domestic livestock losses.

## Introduction

Many zoonoses have environmentally-mediated indirect transmission, where pathogens can persist over time in reservoirs (e.g. on or in soil, grasses, or water), and transmission occurs through ingestion (*e.g*. chronic wasting disease (CWD), *Bacillus anthracis, Brucella spp., Vibrio cholerae*, and *Escherichia coli* 0157:H7) [1–7]. Infected hosts contribute pathogens to these environmental reservoirs via pathogen shedding or host death. Predicting the transmission dynamics in these systems is challenging, as data collection on pathogen persistence, host/pathogen contact, and infection rates are difficult to measure empirically. The compartmental modeling approach has long been used in disease ecology to understand disease dynamics [8–13]. Classical compartmental models focused on directly transmitted diseases in populations can be extended to environmentally-mediated indirect disease transmission [3, 4, 14–16].

Many directly and indirectly transmitted infectious diseases show seasonal patterns in their population dynamics triggered by different intrinsic and extrinsic factors [9, 17]. Disease dynamics can be determined by seasonally pulsed births of the host [18, 19], changes in vector abundance or parasite virulence related to climatic variables [20, 21], or changes in host stress that increase the susceptibility to parasites [22]. In order to effectively control and predict the outcome of disease outbreaks, it is crucial to tease apart the influence of the different factors that determine disease dynamics [23].

Anthrax, caused by the spore forming bacterium *Bacillus anthracis*, is a worldwide zoonosis [2, 24]. The disease is not spread directly from contact between ill and susceptible animals, but via exposure to bacterial spores in the environment [24]. The spores can remain viable in the soil for extended time periods (several years) [25–27], and can infect many species of wildlife and livestock, especially herbivores [24, 25]. The carcass sites of hosts killed by anthrax can become locally infectious zones (LIZs) [28, 29].

Compartmental models have been proposed to understand natural anthrax dynamics in herbivores [14, 15, 21, 30, 31]. The complexity and reality of these models has increased, incorporating animal migration [14] and strong seasonal effects linked to host reproductive cycle [21]. Even though most previous models assume that individuals cannot recover from infection and do not return to the susceptible population [14, 31], some studies have shown that many grazers recover and develop antibody titers against anthrax toxins [28, 32, 33]. A more recent model incorporated pathogen virulence to explain host anthrax resistance [21]. Also, most previous models do not account for seasonal forcing in the infection, although it has been widely suggested that anthrax dynamics are closely tied to environmental drivers that may or may not match the seasonality of host population dynamics [26, 34, 35]. Finally, all models are deterministic in form (*i.e*. [14, 15, 21, 30, 31]) but stochastic models are amenable to realistic predictions regarding disease persistence or extinction [36, 37].

Here we developed deterministic and stochastic models of anthrax transmission in bison (*Bison bison bison*) allowing immune individuals to transition into the susceptible population. We incorporate a stochastic infection probability assuming that the force of infection is seasonal following an environmental covariable. Our model is an adaptation of previously developed models for understanding CWD [4] and anthrax [38] in elk (*Cervus canadensis*) and bison populations in Montana. We investigated the role of population dynamics and seasonal forcing in infection probability on determining outbreak dynamics. We also defined the model parameter space in which the basic reproduction number was different than unity. Finally, we attempted to predict anthrax transmission for a mid-latitude grassland system with ungulate grazers (bison and elk) using maximum likelihood.

## Materials and methods

**Fig 1:**
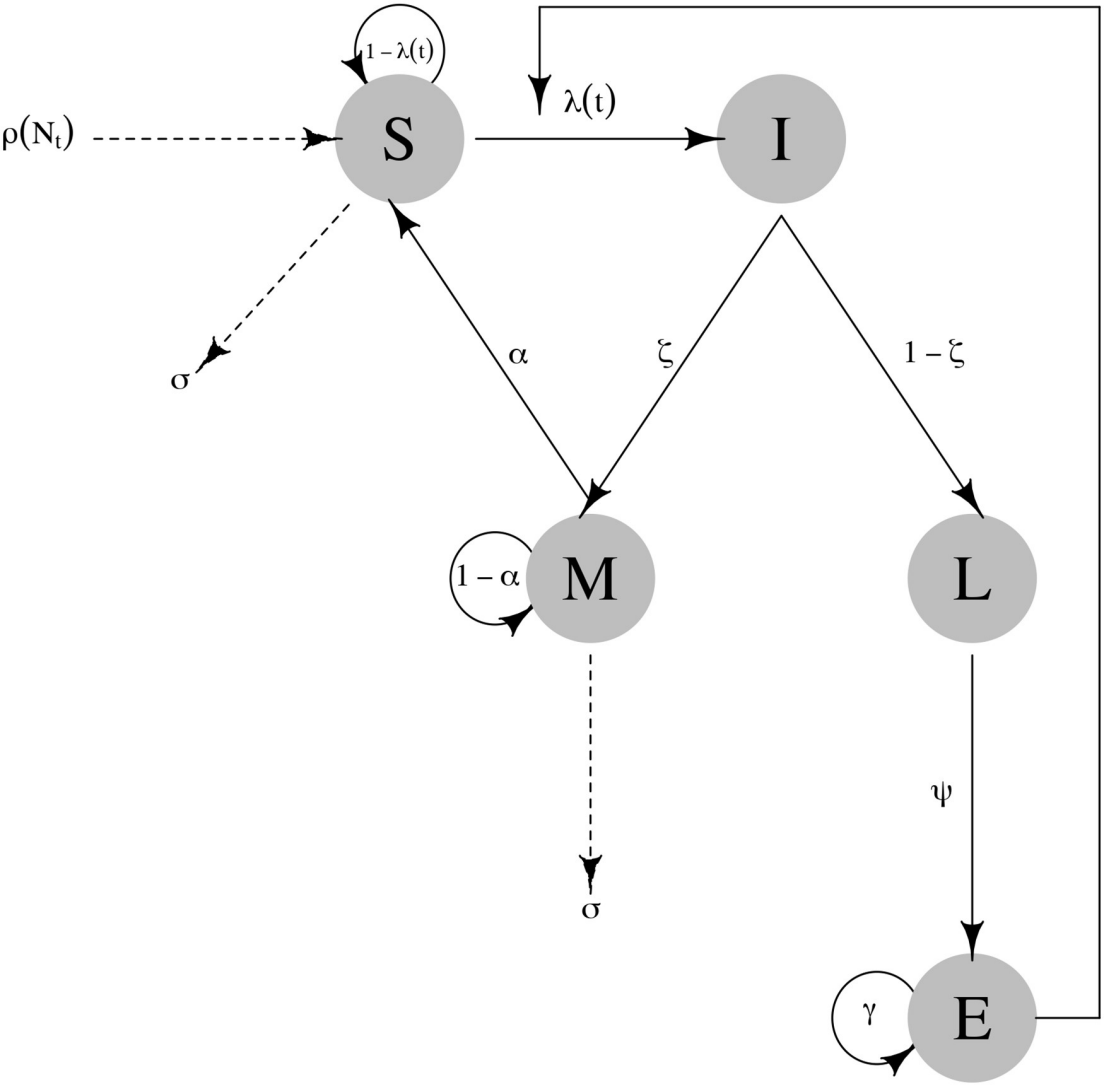
Conceptual diagram of the SMILE model describing compartments and transition probabilities among compartments. The diagram also includes parameters related to population dynamics such as reproduction and non-disease related deaths. Solid lines represent processes that are disease mediate while dashed lines represent non-disease related births and deaths.

The model is based on five compartments that capture various disease states of hosts through death and release of spores into the environment (susceptible (S), immune (M), infected (I), locally infectious zone (L), environment (E); Fig 1). Although previous models were developed using a box-car approach (see [4, 38] for details), to maintain simplicity of the model we do not use any compartment sub-structuring. Using the deterministic basis of the model, we assume that the outcomes of host birth and natural death, infection, immunity, disease death, and spore decay are binomially distributed, to develop the stochastic version. We parameterized our deterministic and stochastic models based on a multi-species outbreak of anthrax, which occurred in southwest Montana where no cases had been reported in decades (Montana outbreak from here on; [7, 39]). Nearly 300 Bison managed as livestock were lost, incurring significant costs from loss of livestock and disease control [7, 33].

### SMILE model description

Overall our model consists on five compartments that describe the weekly anthrax dynamics in bison, but can be easily applied to any system where pathogens are indirectly transmitted through the environment (*e.g*. brucellosis). The susceptible *S* compartment tracked bison that were available for infection in each time step. When individual bison become infected with probability λ they transition into the infected compartment *I*. Infected individuals can either become immune *M* with probability *ζ* or die with probability (1 − *ζ*). The proportion *ζ* of hosts that survive given an exposure to *B. anthracis* in the environment is not well known, but several species develop antibody titers indicating they have survived an exposure [28, 33]. The immune (*M*) compartment held bison that had become immune through spore exposure assuming that antibody titers remain detectable for one year [33, 40]. Each week, immune individuals had a probability *α* of becoming susceptible again or 1 − *α* to remain immune. Those that did not survive infection were assumed to have died of acute anthrax, and transitioned to the LIZ *L* compartment (*i.e*. carcasses infect the environment *E*). Acute illness in bison can lead to rapid death (∼ three days [24]). The *L* compartment counted the number of bison who had died of anthrax in each time step.

The carcass of any anthrax-killed bison was assumed to introduce a specified number of spores *ψ* to the environment *E*. After ingestion from *E*, spores germinate into vegetative cells leading to acute illness and death [24]. After host death, *B. anthracis* vegetative cells sporulate and disseminate around the carcass through decomposition or scavengers [41]. Generally, *E* is the cumulative number of spores in the environment available for infection. *Bacillus anthracis* spores can persist in the environment for extended periods [26, 42, 43] but may lose virulence with probability 1 − *γ* through loss of the pX02 plasmid [2, 24, 44, 45].

Susceptible *S* and immune *M* individuals survive to non-related disease deaths from one time step to another with probability *σ*. At the end of each year, adult bison in *S* and *M* reproduced by a single pulse and each individual reproduces with density-dependent probability [4, 46]. It is rare for individuals younger than one year old to die from anthrax (Blackburn J.K. unpublished data, [32, 47]), but we assumed these individuals entered the system as susceptible *S* and overall calf mortality was similar to adult mortality. The latter assumption will not bias the model since it only increases the influx of individuals to the *S* compartment by approximately 24%, the previously estimated mortality rate of calves during the first year [48, 49].

With the above description, a simple discrete time model where *t* is one week can be written. The set of recursions representing the changes specified above are given by:

$$\begin{array}{cc} S_{t} & =\sigma \left(\left(1-\lambda \right)S_{t-1}\right)+\sigma \left(\alpha M_{t-1}\right),\\ M_{t} & =\zeta I_{t-1}+\sigma \left(\left(1-\alpha \right)M_{t-1}\right),\\ I_{t} & =\lambda S_{t-1},\\ L_{t} & =\left(1-\zeta \right)I_{t-1},\\ E_{t} & =\psi L_{t-1}+\gamma E_{t-1}. \end{array}$$ (1)

This model can be used as the deterministic skeleton of a stochastic formulation. In the stochastic formulation, the demographic events and the transitions from one compartment to another can be modeled with Binomial and Poisson distributions. By using these, the deterministic model above becomes the expected value (mean) of the weekly predictions. The stochastic model we used is written as

$$\begin{array}{cc} S_{t} & ∼Binomial\left(S_{t-1}-I_{t},\sigma \right)+M_{r_{t}}\\ M_{s_{t}} & ∼Binomial(M_{t-1},\sigma )\\ M_{r_{t}} & ∼Binomial(M_{s_{t-1}},\alpha )\\ M_{t} & ∼Binomial\left(I_{t-1},\zeta \right)+\left(M_{s_{t}}-M_{r_{t}}\right)\\ I_{t} & ∼Binomial(S_{t-1},\lambda )\\ L_{t} & ∼I_{t-1}-M_{t},\\ E_{t} & ∼Pois\left(\psi L_{t-1}\right)+Binomial\left(E_{t-1},\gamma \right). \end{array}$$ (2)

We needed to create an extra recursion in the stochastic version for the *M* compartment since the number of Immune individuals recovered ($M_{r_{t}}$) depends on the realization of Immune individuals that survived ($M_{s_{t}}$) from previous time. The most critical component of this model is the term that specifies the force of infection λ. Many plausible functional forms for λ could be used and here we chose to follow the approach of [50] to derive our probability λ.

### First principles modeling of the incidence rate

Ponciano and Capistrán [50] used a general stochastic processes framework to derive the incidence rate function from basic biological principles characterizing epidemic models. They showed that general incidence rate functions result from modeling the number of successful transmission encounters as a pure birth process. They derived an expression for the probability of one or more successful transmission encounters when heterogeneity in the per-individual-transmission probability is taken into account.

In the case of anthrax, where infection is indirect, once an individual host has been infected, that individual will disperse to a particular area where, if it dies, its carcass (LIZ) will infect a given number of susceptible individuals. In practice, secondary dispersion can be neglected since scavengers and other carnivores feeding from the infected carcass only disperse *B. anthracis* spores to a small radius around the carcass. The *disease dispersion effort* can then be thought of as a combination of the distance traveled by the infected individual and the total time the carcass has been on the ground. As time accumulates, successful transmission events also accumulate. Mathematically, the realized dispersion effort could be expressed as a continuous quantity computed from both the distance traveled by the infected animal and the total time since LIZ formation. After death, disease dispersion effort, *a*, can only increase by a quantity Δ*a* proportional to time.

We modeled the total number of visitors to the LIZ that get infected as a pure birth process where the quantity being born is the number of successful transmission events occurring in a LIZ. The number of successful transmission encounters per LIZ will be modeled with a random variable that changes as a function of *a*, *X*(*a*) [50]. To formulate our birth process, we first assumed that the probability that a LIZ infects a susceptible individual given a realized change in dispersion effort Δ*a* (*i.e*. proportional to a small time increment) is proportional to the previous number of successful infections since LIZ formation and to a function of the average density of infectious spores in *E*. This average density of infectious spores is taken to be a measure of the infection potential of the population of LIZs. These assumptions allowed us to specify a new infection event as the conditional probability

$$\begin{array}{c} P[X(a+\Delta a)=x|X(a)=x-1]=\delta (x-1)\Delta aE \end{array}$$ (3)

where *δ*(.) is a non-negative function such that *δ*(0) = *b* is a constant. We remark that by definition, the expected value of *X*(*a*) is equal to the mean number of secondary infections, *R*_0_. Assuming that the probability that more than one successful infectious encounter occurs after an extra dispersion amount Δ*a* is negligible, then *X*(*a*) can be modeled using a simple homogeneous birth process where the quantity being born is the number of successful transmission encounters. The probabilistic law of this stochastic process is completely defined by the terms *p*_*x*_(*a*) ≡ *P*(*X*(*a*) = *x*), *x* = 0, 1, 2, …. To solve for these terms, first note that according to Eq 3

$$\begin{array}{c} p_{x}\left(a+\Delta a\right)=\delta \left(x-1\right)\Delta aEp_{x-1}\left(a\right)+\left[1-\delta \left(x\right)\Delta aE\right]p_{x}\left(a\right), \end{array}$$

which leads to

$$\begin{array}{c} \frac{p_{x}\left(a+\Delta a\right)-p_{x}\left(a\right)}{\Delta a}=\delta \left(x-1\right)Ep_{\left(x-1\right)}\left(a\right)-\delta \left(x\right)Ep_{x}\left(a\right). \end{array}$$

In the limit when Δ*a* → 0, the above equation leads in turn to the following system of differential equations:

$$\begin{array}{c} \frac{dp_{x}\left(a\right)}{da}=E\left[\delta \left(x-1\right)p_{x-1}\left(a\right)-\delta \left(x\right)p_{x}\left(a\right)\right],\;x=0,1,2,3,\ldots \end{array}$$

Then solving this system of equations [51] leads to

$$\begin{array}{cc} p_{0}\left(a\right) & =\exp^{-a\delta (0)E}=\exp^{-abE},\\ p_{x}\left(a\right) & =\exp^{a\delta (x)E}\delta \left(x-1\right)E\int_{0}^{a}\exp^{\delta (x)Es}p_{x-1}\left(s\right)\partial s. \end{array}$$

Furthermore, approximating *δ*(*x*) using a Taylor Series expansion around 0 leads to specific quantitative definitions of the stochastic process *X*(*a*). For example, if *δ*′(0)>0 or if *δ*′(0) = 0, the one-step transition probability density function (pdf) of *X*(*a*) adopts the Negative Binomial and Poisson forms, respectively [52]. In any case, the probability that one LIZ produces one or more infected individuals is

$$\begin{array}{c} P\left(X\left(a\right)\geq 1\right)=1-p_{0}\left(a\right)=1-\exp^{-abE}. \end{array}$$

In a given population however, the magnitude of the realized disease dispersion for each infected individual can be expected to vary widely. To take into account this demographic source of heterogeneity, we model variation in disease dispersion assuming that *a* is a random variable whose pdf *f*_*B*_(*a*) has support on (0, ∞). Then, the probability that an infected individual chosen at random from the population realizes more than one successful secondary infection is found by averaging 1 − exp^−*abE*^ over all the possible realizations of *a*. That is,

$$\begin{array}{c} P\left(X\left(a\right)\geq 1\right)=\int_{0}^{\infty }(1-\exp^{-abE})f_{B}\left(a\right)da. \end{array}$$

A probabilistic model for *a* that has empirical and theoretical support in the genetics literature of modeling fitness distributions is the exponential model [53]. However, the exponential model is just a special case of the gamma distribution with shape parameter equals to one. To allow more flexibility in dispersal heterogeneity, we assumed that *a* followed a gamma distribution with shape and rate parameters *θ* and *τ* respectively. The assumption behind using the gamma distribution here is the magnitude of the disease dispersion brought about by an infected individual is proportional to fitness. Accordingly, letting $f_{B}\left(a\right)=\frac{\theta^{\tau }}{\Gamma (\tau )}a^{\tau -1}e^{-\theta a},\;0<a<\infty$ we get the probability of successfully transmission is

$$\begin{array}{c} P\left(X\left(a\right)\geq 1\right)=\int_{0}^{\infty }(1-\exp^{-abE})\frac{\theta^{\tau }}{\Gamma (\tau )}a^{\tau -1}e^{-\theta a}da. \end{array}$$

When *δ*′(0) = 0, the probability that a LIZ causes a new infection does not depend on previous cases. Then, the probability mass function (pmf) of *X*(*a*) is Poisson [52]. Taking into account the heterogeneity in dispersion then amounts to integrating this pmf over the distribution of *a*, *i.e*.,

$$\begin{array}{c} P\left(X\left(a\right)=x\right)=\int_{0}^{\infty }\frac{e^{-abE}abE^{x}}{x!}\frac{\theta^{\tau }}{\Gamma (\tau )}a^{\tau -1}e^{-\theta a}da, \end{array}$$

which upon integration gives (See S1 Appendix)

P(X=x)=(x+τ-1x)(θbE+θ)τ(bEbE+θ)x.

Then, we immediately get that

$$\begin{array}{c} P\left(X=0\right)={\left(\frac{\theta }{bE+\theta }\right)}^{\tau },\;\text{and}\;\text{that} \end{array}$$

$$\begin{array}{c} \lambda \left(t\right)=P\left(X\geq 1\right)=\frac{{\left(bE+\theta \right)}^{\tau }-\theta^{\tau }}{{\left(bE+\theta \right)}^{\tau }}. \end{array}$$ (4)

This expression for the infection probability depends on the distribution of the dispersion effort (*a* ∼ Gamma(*θ*, *τ*)), the number of infections that a LIZ causes assuming no dispersion effort (*b*), and the number of spores in the environment (*E*). One of our primary objectives was to identify the possibility that anthrax disease dynamics are a result of an external seasonal driver causing seasonal forcing in the infection. To account for the latter possibility we assumed *b* to vary seasonally by assuming that *b* is a sinusoidal function of time (*t*)

$$\begin{array}{c} b\left(t\right)=e^{\left(b_{0}(1+b_{1}(\text{cos}(\frac{2\pi t}{\Pi })))\right)}, \end{array}$$ (5)

where *b*_0_ and *b*_1_ determine the strength of the seasonality and *Π* is the outbreak periodicity. Other scenarios exist in which the probability of a new infection either decreases or increases with the number of previous infections that a LIZ has produced. This accounts for the cases in which *δ*(*x*) = *b* + *cx* and *δ*(*x*) = *b* − *cx*.

From this form of infection probability we can obtain a local *R*_0_, the number of cases that a single LIZ can cause. Because the process *X* counts the number of successful transmissions of a single carcass introduced in a population of non-infected individuals (after accounting for heterogeneity in dispersion), then its expected value E[X] can be thought of as the mean number of secondary infections or *R*_0_ in the context of this disease transmission setting. For the case in which the probability that a specific LIZ produces an additional infection does not depend on previous infections, E[X] is simply the expected value of a Negative Binomial distribution with parameters *τ* and probability $\frac{bE}{bE+\tau }$ (See S1 Appendix). Thus, the local *R*_0_ for this specific model is

$$\begin{array}{c} E\left[X\right]=R_{0}=\frac{\tau \text{bE}}{\theta }. \end{array}$$ (6)

We derive the infection probability and *R*_0_ for the cases in which *δ*(*x*) = *b* + *cx* and *δ*(*x*) = *b* − *cx* in S1 Appendix.

### Simulations

We developed a simulation experiment to determine the effect of population dynamics and seasonal forcing in model predictions. The first model ignored both population and seasonal dynamics. We assumed no reproduction (the addition of new susceptible individuals from reproduction) and no death of individuals other than disease related. Infection probability in this model was taken as in Eq 4 with constant *b*. Next, we allowed *b* to vary seasonally as described in Eq 5. Following, we allowed individuals to reproduce annually with density dependent reproduction, in which every individual had a probability of reproduction according to Eq 7 and constructed models with and without seasonal dynamics.

For all models we assumed *θ* = 10, *τ* = 10 and allowed them to run for ten years (520 weeks). For the cases with no seasonal dynamics we assumed *b* = 0.001 and for the cases with seasonal forcing we assumed *b*_0_ = −30 and *b*_1_ = 0.85. For all cases, initial population size was 3500 which was the approximate bison population size during the 2008 Montana outbreak. In the models with population dynamics, reproduction was estimated as a single year-end pulse assuming density-dependent probability of reproduction [4, 46]

$$\begin{array}{c} \rho (N_{t})=\frac{\rho }{1+{\left(\frac{N_{t}}{K}\right)}^{10}} \end{array}$$ (7)

where *ρ* was the averaged reproduction rate, *N* is the total population, and *K* is the carrying capacity, and the exponent is a factor that affects how dependent reproduction is to density [46]. Only susceptible or immune bison were included in the birth-eligible population. We estimated *ρ* = 0.41 from literature on bison herds in Yellowstone and elsewhere in North America and fixed *K* = 5000 [46–48, 54, 55].

In [32], up to 70% of wood bison, *B. bison athabascae*, had high titers after an epizootic event. Given the 172 deaths in the latter event of a population of 2026 individuals, this implies that approximately 88% of exposed bison survived with high titers. We used this as an estimate of probability of bison surviving spore exposure and developing immunity (*ζ*). The quantity of spores released (*ψ*) is uncertain and for computational ease, a unit-less value of 1 was used. Given the uncertainty of how long spores remain viable and virulent in the environment, we assumed that spores decay at the highest rate possible (*γ*_1_ = 0.0132) as a conservative estimate [38].

Finally, we explored the parameter space to determine the conditions under which *R*_0_ was different from unity. Specifically, we wanted to know what conditions of dispersal could increase or decrease disease persistence. We allowed both *θ* and *τ* to vary between 0.01 and 100 and assumed seasonal forcing as above. We selected values of *b* that were high (*b* = 0.01), intermediate (*b* = 0.005) and low (*b* = 0.0001), characteristic of the peak of an outbreak, midway before/after an outbreak and between outbreaks. Since the *R*_0_ is also dependent on the number of spores in the environment (*E* in Fig 1; see Eq 6), we fixed the number of spores to the highest possible obtained in one of the simulations (*i.e*. 800; see Table 1 for a summary of all the parameters of the SMILE model and the values used for simulations).

**Table 1:** Parameters considered in the SMILE model and their description. The values described in the Value column correspond to the values used during simulations and in the cases in which the parameters are functions, the equation is given.

Parameter	Description	Value
λ(*t*)	Force of Infection	$P\left(X\left(a\right)\geq 1\right)=\frac{{\left(bE+\theta \right)}^{\tau }-\theta^{\tau }}{{\left(bE+\theta \right)}^{\tau }}$
*τ*, *θ*	Define dispersion effort	*τ* = 10, *θ* = 10
*b*	Number of infections caused by one LIZ when $\frac{\tau }{\theta }=0$	0.001
*b*_0_, *b*_1_	Strength of Seasonality	*b*_0_ = −30, *b*_1_ = 0.85
Π	Periodicity of infection	3 years
*α*	Probability of transitioning from Immune to Suceptible	0.02
*ζ*	Probability of becoming Immune after Infection	0.88
*ψ*	Number of Spores per carcass	1
*γ*	Spore persistence rate	0.9868
*ρ*(*N*_*t*_)	Reproduction Probability	$\rho (N_{t})=\frac{\rho }{1+{\left(\frac{N_{t}}{K}\right)}^{10}}$
*N*_*t*_	Population size at time *t*	*S*_*t*_ + *M*_*t*_
*ρ*	Average Reproduction Rate	0.41
*K*	Carrying capacity	5000

### Bias and parameter estimation

We designed an additional simulation experiment to evaluate the bias in the estimation of the parameters. Because of the complexity of the stochastic model it was difficult to derive the likelihood function for each of the time series. Instead, we assumed that observed time series in the field might be the product of observation error. Thus, we performed parameter estimation using the deterministic model and assuming observation error as a Poisson realization of the deterministic model. For example, if *L*_*t*_ and *l*_*t*_ are the expected and observed number of carcasses at time *t* respectively, then *l*_*t*_ ∼ Poisson(L_t_). We assumed the same rationale for all of the other time series in the model (*i.e*. S, M, I, E).

We generated 100 data sets using the stochastic version of SMILE allowing it to run for 10 years fixing *θ* = 10, *τ* = 10, *b*_0_ = −30 and *b*_1_ = 0.85, assumed a disease periodicity (Π) of three years and left the compartment transition probabilities and population dynamics parameters as described in previous section. We estimated the values of *b*_0_, *b*_1_, *τ* and *θ* that maximized

$$\begin{array}{c} \begin{array}{cc} \text{ln}(L(s,m,i,l,e;b_{0},b_{1},\tau ,\theta )) & =\sum_{t=1}^{T}\frac{S_{t}^{s_{t}}e^{-S_{t}}}{s_{t}!}+\sum_{t=1}^{T}\frac{M_{t}^{m_{t}}e^{-M_{t}}}{m_{t}!}+\\ & \sum_{t=1}^{T}\frac{I_{t}^{i_{t}}e^{-I_{t}}}{i_{t}!}+\sum_{t=1}^{T}\frac{L_{t}^{l_{t}}e^{-L_{t}}}{l_{t}!}+\sum_{t=1}^{T}\frac{E_{t}^{e_{t}}e^{-E_{t}}}{e_{t}!} \end{array} \end{array}$$ (8)

for each of the 100 stochastic data sets. While the parameters do not directly define the likelihood function, they are required to generate the deterministic prediction of the model. We then calculated the bias in each of the parameters as the relative deviation from the true value. The relative bias for *b*_0_ is $\text{Bias}\left(b_{0}\right)=\frac{\hat{b_{0}}-b_{0}}{b_{0}}$, for example. We estimated the bias in the parameters assuming that all the SMILE time series were available, a subset of them where available (i.e. SMIL, SML, SL and L) and also by removing one time series at a time and all possible combinations of two time series. Estimation of the parameters was performed by minimizing the negative log likelihood in the optim function in R [56]. The code used for parameter estimation is available online.

### Prediction of the 2008 Montana outbreak

To further test model performance, we used a one-year real data set from an anthrax outbreak in bison in Montana. The data set consisted of the number of bison deaths each week of 2008. We used the same rationale described above and estimated parameters that maximized Eq 8 given the observed time series. One caveat in the real scenario was the limit of a single year of data, it was impossible to estimate periodicity of additional outbreaks. To evaluate the effect of the period on the prediction of the outbreak, we used a likelihood profile approach. We used 100 values of *T* varying between 1 and 20 and for each of the values estimated the values of *b*_0_, *b*_1_, *τ*, and *θ* that maximized the value of Eq 8. We then retained the value of the *T* that yielded the maximum likelihood.

## Results

### Simulations

**Fig 2:**
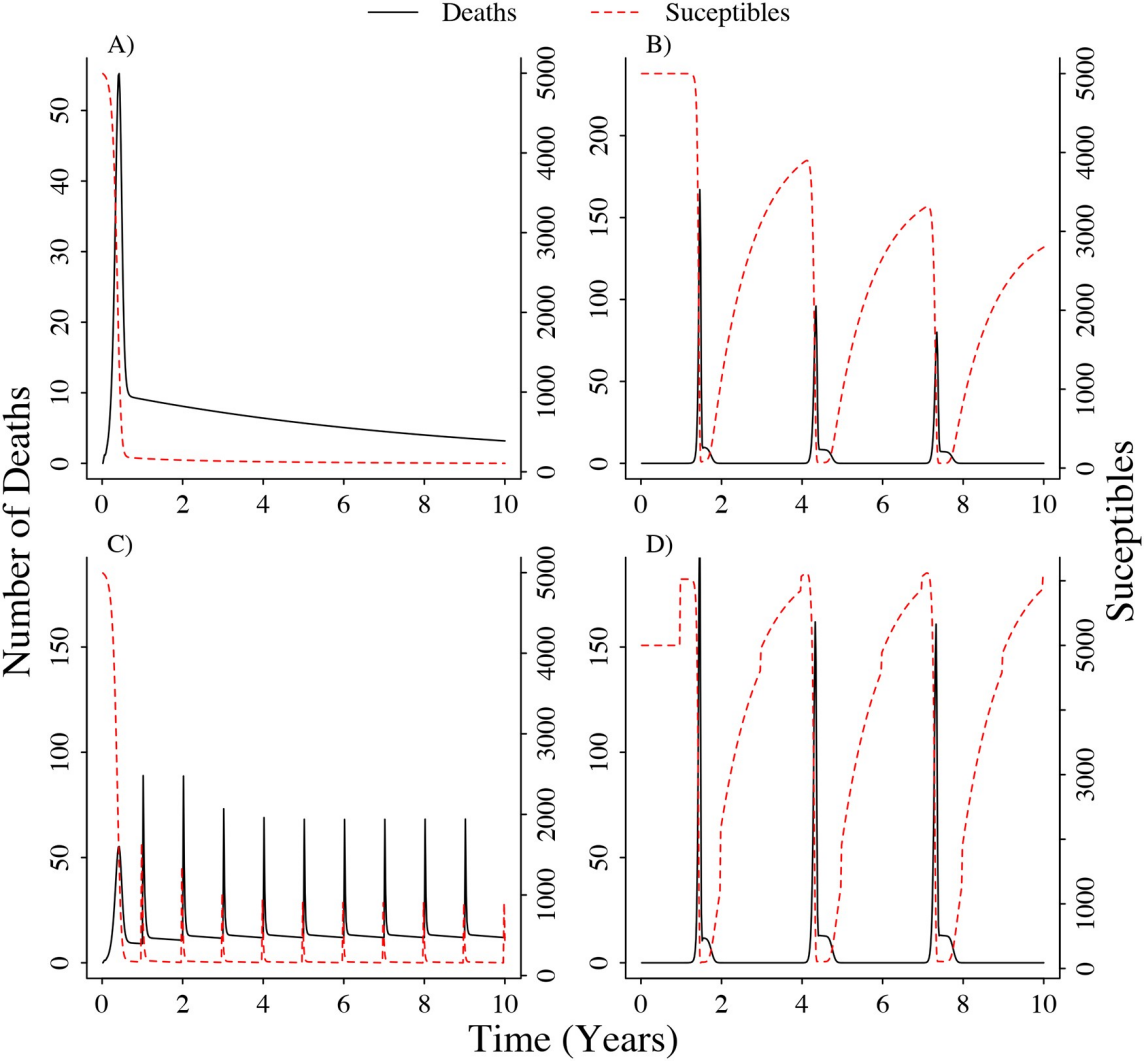
Different types of simulations showing the effect of host population dynamics and seasonal driver on the number of deaths caused by *Bacillus anthracis*. A) Simulations without population dynamics and seasonal dynamics B) Simulations without population dynamics C) Simulations without seasonal infection dynamics D) Simulations with population and seasonal dynamics. For all cases we assumed *τ* = 10, *θ* = 10 and for simulations with seasonal infection we set *b*_0_ = −30, *b*_1_ = −0.85 and the period was three years.

The simplest model that we tested, which did not take into account population dynamics or seasonality in the infection probability, showed an increase at the beginning of the simulation that correlated with the decrease of the susceptible population. Once most susceptibles were depleted, the number of anthrax deaths decreased until nearly zero cases were observed after the ten year period (Fig 2A). Introducing seasonality in the infection probability had the effect of producing three disease outbreaks during the ten year period, matching the periodicity fixed to *b* in the simulations. Although there is no addition of susceptible individuals to the population through reproduction, decreasing the infection probability during endemic years allows immune individuals to recover and be available for infection during the following outbreak season (Fig 2B).

Introducing population dynamics, such as reproduction and deaths by causes other than disease related, produced a pattern of a disease outbreak every year following a reproduction event (Fig 2C). When incorporating both population and seasonal dynamics, the model showed a similar pattern as without population dynamics. Three disease outbreaks were observed, determined by the seasonality in the infection probability and with little effect from reproduction events. Susceptibles are replenished by the recovery of immune individuals and reproduction events during the endemic years (Fig 2D). During the peak of the outbreak, *R*_0_ was greater than 1 in the cases in which *θ* ≤ 8*τ* (S1A Fig). During the time the outbreak was midway, *R*_0_ > 1 for cases in which *θ* ≤ 6*τ* (S1B Fig). Finally, during endemic years, *R*_0_ was only larger than one in cases in which *θ* ≤ 0.08*τ* (S1C Fig).

### Bias and parameter estimation

**Fig 3:**
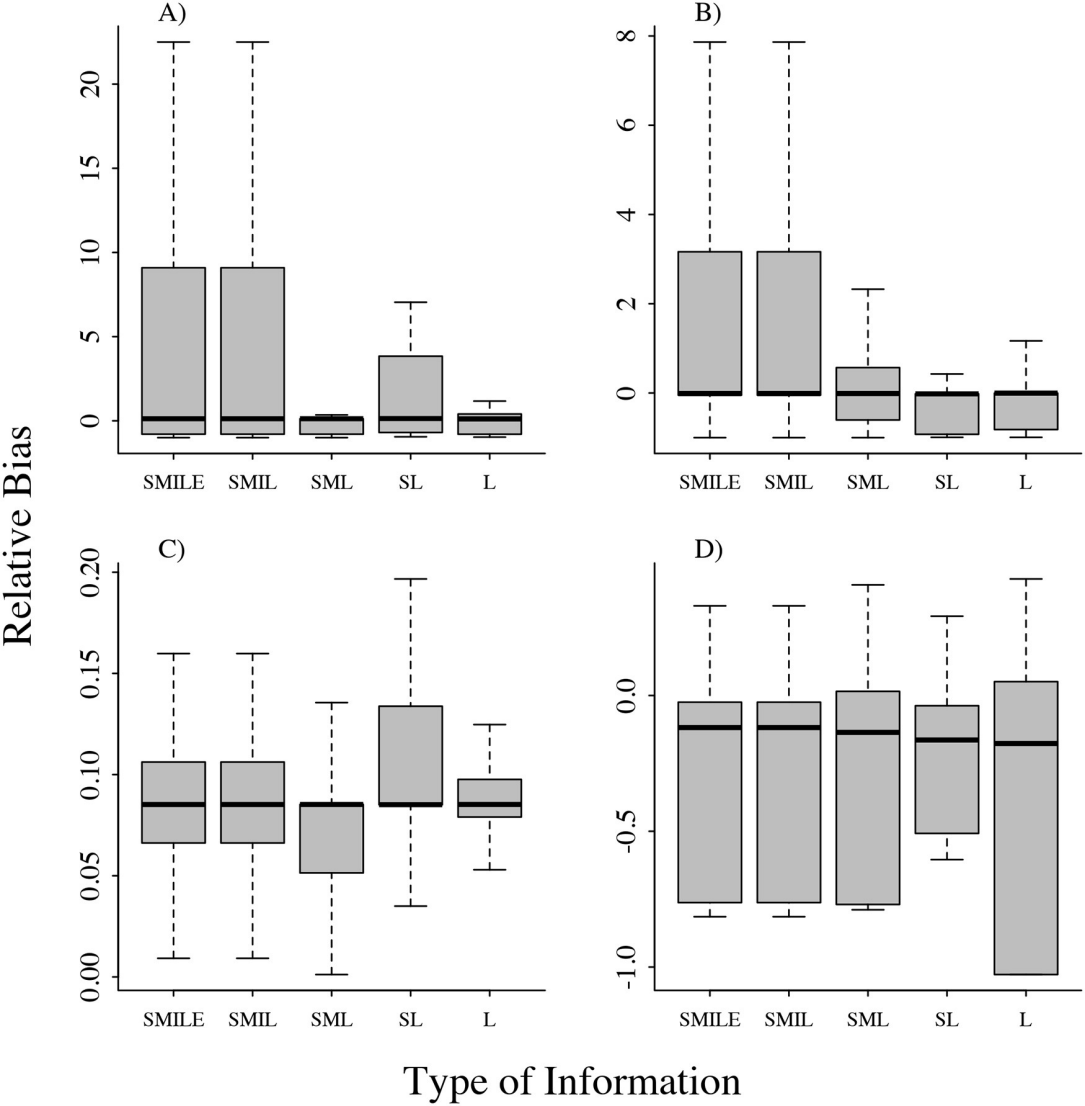
Relative bias in the estimation of the parameters *τ*, *θ*, *b*_0_ and *b*_1_ different amount of information. Labels in the *x* axis refer to the time series used for estimation. A) *τ*, B) *θ*, C) *b*_0_, D) *b*_1_. SMILE: Susceptible, Immune, Infected, LIZ, Environment; SMIL: Susceptible, Immune, Infected, LIZ; SML: Susceptible, Immune and LIZ; SL: Susceptible and LIZ; L: LIZ.

Overall, we observed low bias irrespective of the amount of information used for parameter estimation (Fig 3, S2 and S3 Figs). We found that the bias of *θ* and *τ* varied the most when using a larger amount of information (*i.e*. four or five time series from the compartments; Fig 3). We found a small bias in *b*_0_ and *b*_1_. While the estimation tends to overestimate the values of *b*_0_, the values of *b*_1_ were underestimated from 5 to 7%. Bias patterns were also similar independent of the number of time series used (Fig 3, S2 and S3 Figs).

### Prediction of Montana 2008 outbreak

**Fig 4:**
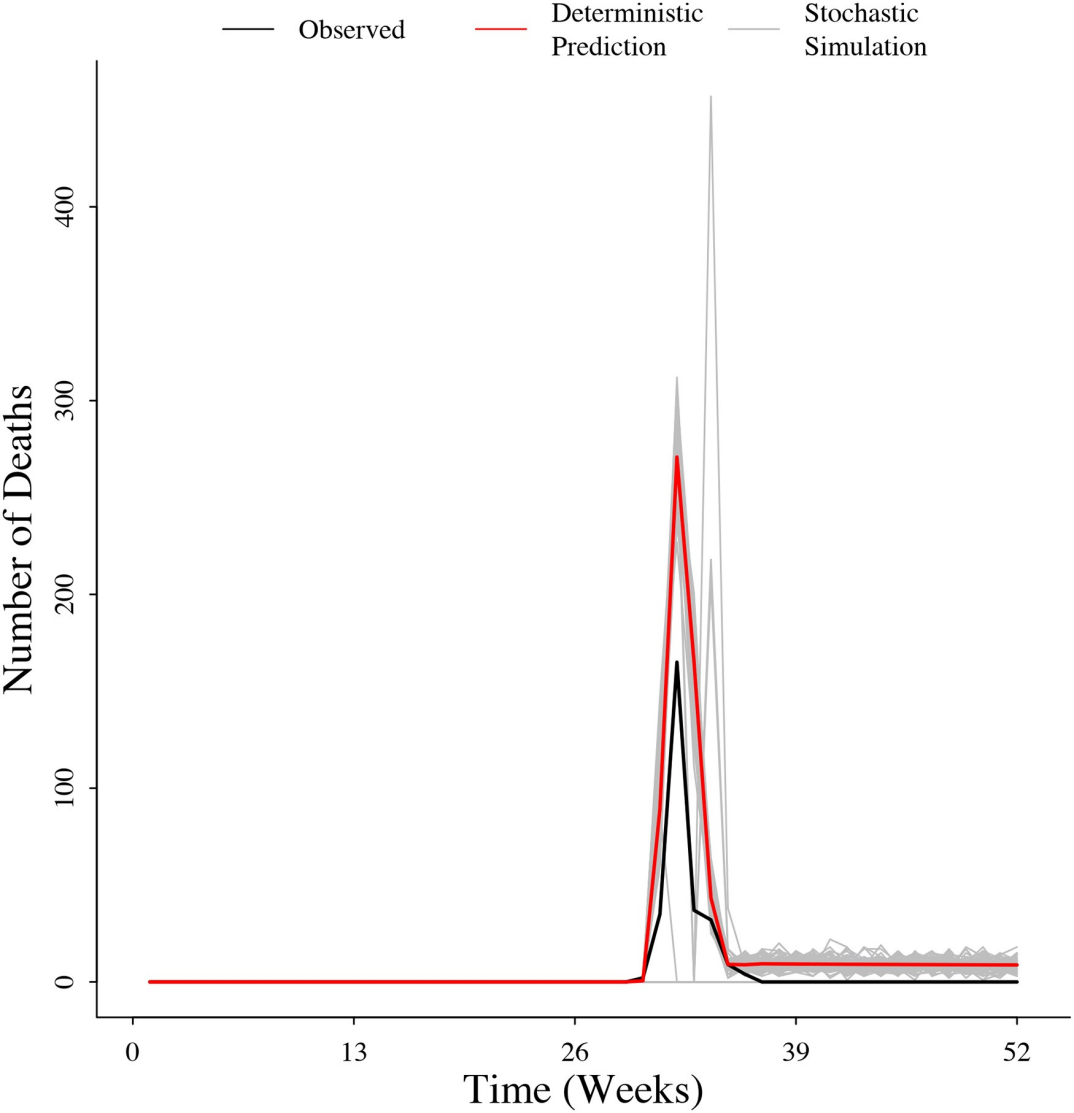
Observed number of cases during the 2008 Montana outbreak and the deterministic and stochastic predictions of the SMILE model.

Using the data from the Montana outbreak, we were able to estimate the parameters and correctly predict the timing of the outbreak (Fig 4). We found *θ* = 927.9, *τ* = 0.12, *b*_0_ = −25.9, and *b*_1_ = 3.9. We found the value of *T* that maximized the likelihood of the model given the Montana outbreak data was *T* = 1.96. Since the model we used to predict the outbreak assumed seasonal forcing on infection dynamics, this means that *R*_0_ was also seasonally larger than one beginning in week 32 and smaller than one between weeks one and 31.

## Discussion

Here we attempted to improve our understanding of the dynamics of environmentally-mediated diseases using a combination of simulation and statistical inference for stochastic processes. Our primary objective was to disentangle the role of population dynamics and environmental seasonality on environmentally-mediated diseases. We demonstrated how for anthrax, assuming a non-seasonal infection probability predicts significant outbreaks yearly after reproduction pulses. From our previous knowledge of anthrax dynamics in mid-latitude grasslands, we know that large outbreaks do not occur annually but instead their periodicity and intensity are determined by specific environmental conditions [26, 34, 35]. Our simulations recreated this scenario. By incorporating population dynamics and allowing seasonal forcing of infection to be dependent on an external factor we estimated seasonality to have a large impact on the number of anthrax-related deaths (Fig 2). Additionally, we identified three specific mechanisms through which *R*_0_ arises, allowing us to estimate scenarios in which *R*_0_ > 1 depending on different epizootic stages through the years.

Early models of anthrax dynamics focused on single year outbreaks and ignored population dynamics [15, 30]. Although useful to understand single epizootic events, these models do not allow prediction to future events because they ignore how populations behave in the absence of the disease. More recent studies have extended such models to incorporate population dynamics and migration in order to make inferences about the persistence of animal populations subject to anthrax [14]. Friedman and Yakubu [14] showed how epizootic events can be maintained in a region by migration of infected individuals into the region. We know however, that this might not be likely in mid-latitude grasslands where the epizootics in specific regions occur without reference to epizootics in other regions. In this sense, it is more likely that a scenario in which environmental variables (*e.g*. precipitation, speed of spring greenup) determine the infectiousness of the LIZs on the landscape triggering outbreaks [35]. In this context, three processes might lead to increased infectiousness: 1) increase or shift in host foraging, 2) mechanical transportation of bacterial spores to the surface or directly on to vegetation or 3) promotion of germination, increasing the pathogen population at the LIZ. We have recreated this infection scenario by adding the seasonal function to the infection probability that depends only on external factors and not on parameters of the disease itself. Further studies can help us identify the specific mechanisms through which infectiousness changes.

Recent anthrax models have demonstrated the conditions for the dynamics to be stable while allowing for a reproductive number larger than unity [31]. It has been shown that the only way in which the disease persists (*i.e*. *R*_0_ > 1) is by having a time delay between infection and death of the individual [31]. We have incorporated the same concept by using an alternative approach to modeling the infection dynamics. We assume that the infection is based on a stochastic birth process but further assume that the dispersion effort is also stochastic, representing variability in the time delay between infection and death. We demonstrate how in different points of the dynamics, *R*_0_ might be larger or smaller than one depending on the dispersion effort. This dispersion effort can alternatively be interpreted as the fitness of the infectious agent itself. Our results show that during epizootic years, even bacteria with very low fitness can contribute substantially to the dynamics by increasing *R*_0_ to values larger than one (S1A and S1B Fig). In contrast, during enzootic years, the persistence of the disease is determined only by bacteria or genetic lineages that have a high fitness (*i.e*. high dispersion effort; S1C Fig).

Outbreaks of environmentally mediated diseases are connected to strong and extreme variablity in climate. For example, some of these outbreaks happen during years experiencing El Nino Southern Oscillation (ENSO) events [57]. The periodicity of these climatic events produce seasonal outbreaks in diseases that are separated by long periods of time, such as the periodicity observed in anthrax in North America. For example, warmer water and air temperature during ENSO events have been suggested as important drivers of the occurrence of cholera in Asia and South America [57]. The shift in the basic reproductive number driven by disease fitness found in our modeling approach can be directly applicable to these other diseases. In years when climatic conditions are suitable for parasite reproduction (i.e. during ENSO years), disease cases are drastically increased by the capability of low fitness parasites to successfully cause an infection. In the light of climate change these extreme climatic events are becoming more frequent, consequently understanding how the transmission of these diseases operates allows for timely control and prevention of outbreaks.

Previous models for anthrax assumed that the only fate of infected individuals was death and posterior contribution of spores to the environment. Our model instead, following on recently published models [21] and literature that suggests that a large proportion of the infected individuals survive [28, 32, 33, 40], allows for a compartment in which individuals acquire immunity for at least one year and then recover. The Immune *M* compartment clearly has a strong impact in driving dynamics as it allows for the susceptible population to replenish without reproduction (Fig 2). Although our model does not allow for variation in infectiousness among LIZs as in [21], we also conclude that disease persistence depends on the variability in the infectiousness of the LIZ [21]. We however show an alternative approach in which the variability is driven inter-annually by external abiotic factors. We still need further studies to understand the potential mechanisms driving this inter-annual variability in infectiousness that might be responsible for the large and sporadic outbreaks observed in the mid-latitude grasslands.

It is interesting to show how three different processes through which LIZs contribute to future infections arrive at the same infection probability (λ). As it is now routinely recognized in the mathematical modeling of diseases literature, from a single deterministic system of equations one can derive multiple *R*_0_ forms [58]. In these cases, the onus falls on the biological interpretability of each mathematical form [58]. We believe that in order to select an adequate *R*_0_ formulation future research needs to be focused on generating data tracking the individual fate of a LIZ and actual accumulation over time of host contact and infection rates per LIZ. To date, obtaining such data remains implausible and logistically challenging.

Although we didn’t develop a likelihood function to find the maximum likelihood estimates under the stochastic version of the model, assuming that the observed data resulted from observation error rather than process error, we still had good parameter estimates irrespective of the amount of data used (Fig 3). Other computer intensive alternatives are feasible but are beyond the scope of this analysis. The parameter estimates resulting from the 100 stochastic simulations showed little to no bias, allowing us to estimate the parameters related to infection probability with confidence. Anthrax is often underestimated and under reported across its range [24]. At the same time, identifying and sampling carcasses in remote wildlife areas remains logistically challenging [59]. Fortunately, we were able to accurately recover the parameters describing the dynamics using only the number of carcasses in the environment at a given week, which is one of the more frequently reported types of data across anthrax literature.

Taking advantage of model properties, we predicted an outbreak in Montana’s mid-latitude grasslands. Our estimation closely matched the observed 2008 outbreak progression in timing and intensity. We successfully recreated the trajectory of the outbreak and estimated dispersion effort. Our model predicted anthrax in this system has a small dispersion effort (i.e. $\frac{\tau }{\theta }=0.0001$), allowing us to conclude that the infection is entirely driven by a change in the environmental variables. This is just the first attempt to estimate parameters, as the outbreak was limited to a single year. Ideally, a longer time series should provide more accurate information on the drivers and shape of the seasonal component especially in predicting the number of deaths.

The apparent overestimation of the number of deaths caused by the outbreak in 2008 might be explained by the stochastic nature of the process. The observed trajectory was conceived as a single stochastic realization of the true process. The deterministic prediction denotes the average size of the epizootic. There are stochastic simulations in which the disease does not develop and some others in which the number of deaths is higher than the expected. The deterministic prediction gives the mean value of the process but the variance is as an important metric in the prediction of outbreaks.

Modeling of a complex system requires abstraction, and a trade-off between including more details and the simplicity of the model [28]. We endeavored to pick the main properties of anthrax that were most relevant to this study, but there are many additional aspects that could be added in the light of additional hypotheses. For example, we did not include any disease interventions, either surveillance and decontamination of carcasses or vaccination strategies. Including vaccination rates and vaccine efficacy in future models could provide useful insight for disease management. No sex structure was included, however, it has been observed in multiple outbreaks, including the Montana outbreak, that male bison have disproportionately high death rates [33, 47, 60–63]. Our model only included indirect transmission from the environment, but it has been seen in some ecosystems that mechanical vectors (such as flies) play a role in anthrax transmission [24, 64]. Finally, hosts of many of these environmentally mediated diseases are migrating organisms or structured in space. Also, there is evidence that grazers are attracted to high infectious zones creating a non-homogeneous distribution of disease in space. Consequently, a component making the model spatially explicit could enhance the predictions of the model and allow us to understand which are the focal areas in which outbreaks are more likely to happen.

Our focus here was anthrax, an exemplar disease with long-term pathogen persistence due to the spore-forming life history strategy of *B. anthracis*. However, our model is not anthrax specific. Our parameters (*θ* and *τ*) can be interpreted in the light of the life history strategy of any environmental pathogen. For example, *Brucella abortus* has been shown to persist for more than 50 days under certain environmental conditions on the Montana landscape [6]. Inter-specific transmission (e.g. elk to domestic cattle) is hypothesized to occur when cattle ingest bacteria aborted or shed during birthing events [5]. In this case, the dispersion effort can be interpreted as the distance moved by an individual from the place of infection to the place of shedding or abortion. In this sense, changing the parameters that determine the population dynamics of the host should be sufficient to test our model on different environmentally-mediated diseases.

## Conclusion

In this study, we provide a mathematical framework and compartmental model for examining the roles of LIZs for indirect transmission where a host contacts the pathogen directly within the environment. Our model provides general knowledge of environmentally mediated diseases by explicitly elucidating how intense environmental events determine the tempo and amplitude of outbreaks of rare diseases. In the light of climate change, these environmental events are prone to increase in frequency and intensity. A solid understanding of the relationship of these events with the frequency and intensity of outbreaks should be useful in aiding prevention strategies of environmentally mediated diseases, including those that are not well understood.

## Supporting information

S1 Fig Variability in *R*_0_ with respect to *τ*, *θ* and A) high, B) intermediate and C) low values of *b* determined by the seasonal component of the infection probability. The white line indicates the one to one ration between *τ* and *θ*. Values above and below the line indicate low and high dispersal effort respectively. Values along the line indicate cases in which mean dispersal effort is one. The inset in each panel shows the trajectory of *b* over time defining the seasonality in the outbreaks. The value of *b* used for calculation of *R*_0_ in each of the three cases is represented by the dashed line in each of the insets.
(TIFF)

S2 Fig Relative bias in the estimation of the parameters *τ*, *θ*, *b*_0_ and *b*_1_ removing one time series at a time. Labels in the *x* axis refer to the time series used for estimation. A) *τ*, B) *θ*, C) *b*_0_, D) *b*_1_. SMILE: Susceptible, Immune, Infected, LIZ, Environment.
(TIFF)

S3 Fig Relative bias in the estimation of the parameters *τ*, *θ*, *b*_0_ and *b*_1_ removing two time series at a time. Labels in the *x* axis refer to the time series used for estimation. A) *τ*, B) *θ*, C) *b*_0_, D) *b*_1_. SMILE: Susceptible, Immune, Infected, LIZ, Environment.
(TIFF)

S1 Appendix Demonstration of the derivation of infection probability as a stochastic process with heterogeneity in dispersion effort. (PDF)

S2 Appendix Observed number of cases during the 2008 Montana anthrax outbreak. Data is presented on a daily basis and only for the range of weeks for which there was at least one anthrax case reported. Date is in Month/Day/Year format.
(PDF)
