# Supplementary figures and images for: Decoupling environmental effects and host population dynamics for anthrax, a classic reservoir-driven disease
Source: PLoS One. 2018 Dec 12;13(12):e0208621. doi: 10.1371/journal.pone.0208621 (PMC6291251; doi:10.1371/journal.pone.0208621)

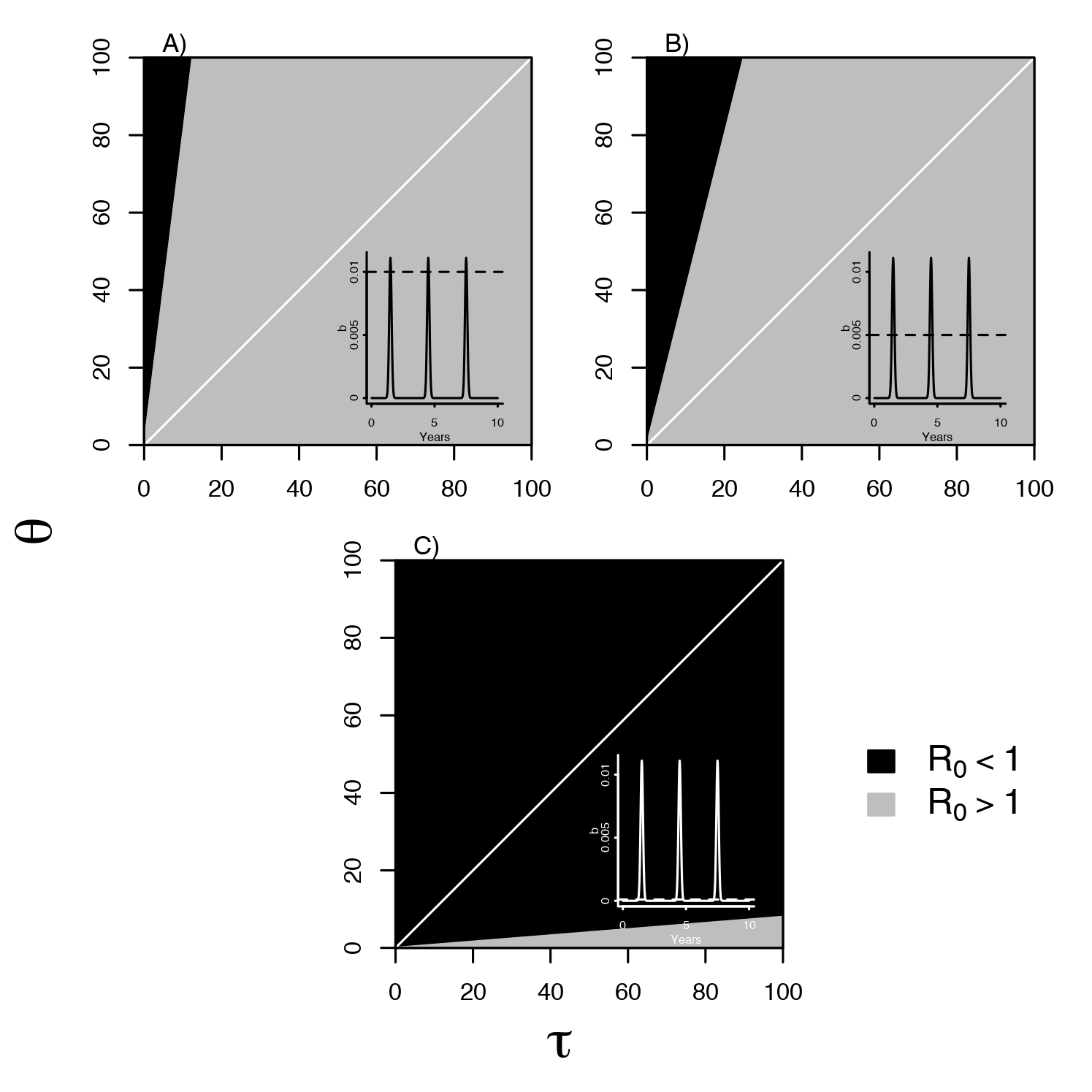

Supplement: S1 Fig — The white line indicates the one to one ration between τ and θ. Values above and below the line indicate low and high dispersal effort respectively. Values along the line indicate cases in which mean dispersal effort is one. The inset in each panel shows the trajectory of b over time defining the seasonality in the outbreaks. The value of b used for calculation of R0 in each of the three cases is represented by the dashed line in each of the insets. (TIFF) [file pone.0208621.s001.tiff]

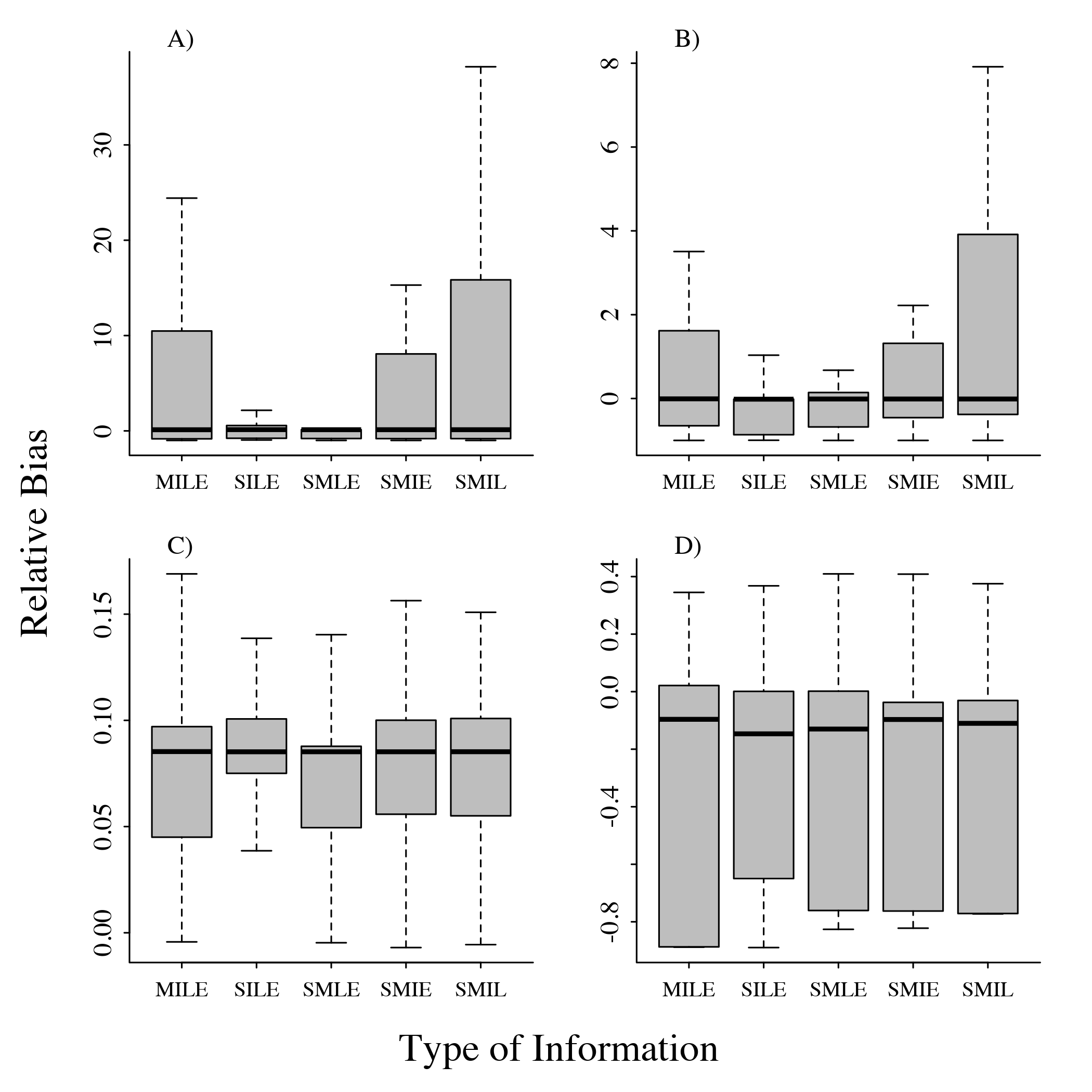

Supplement: S2 Fig — Labels in the x axis refer to the time series used for estimation. A) τ, B) θ, C) b0, D) b1. SMILE: Susceptible, Immune, Infected, LIZ, Environment. (TIFF) [file pone.0208621.s002.tiff]

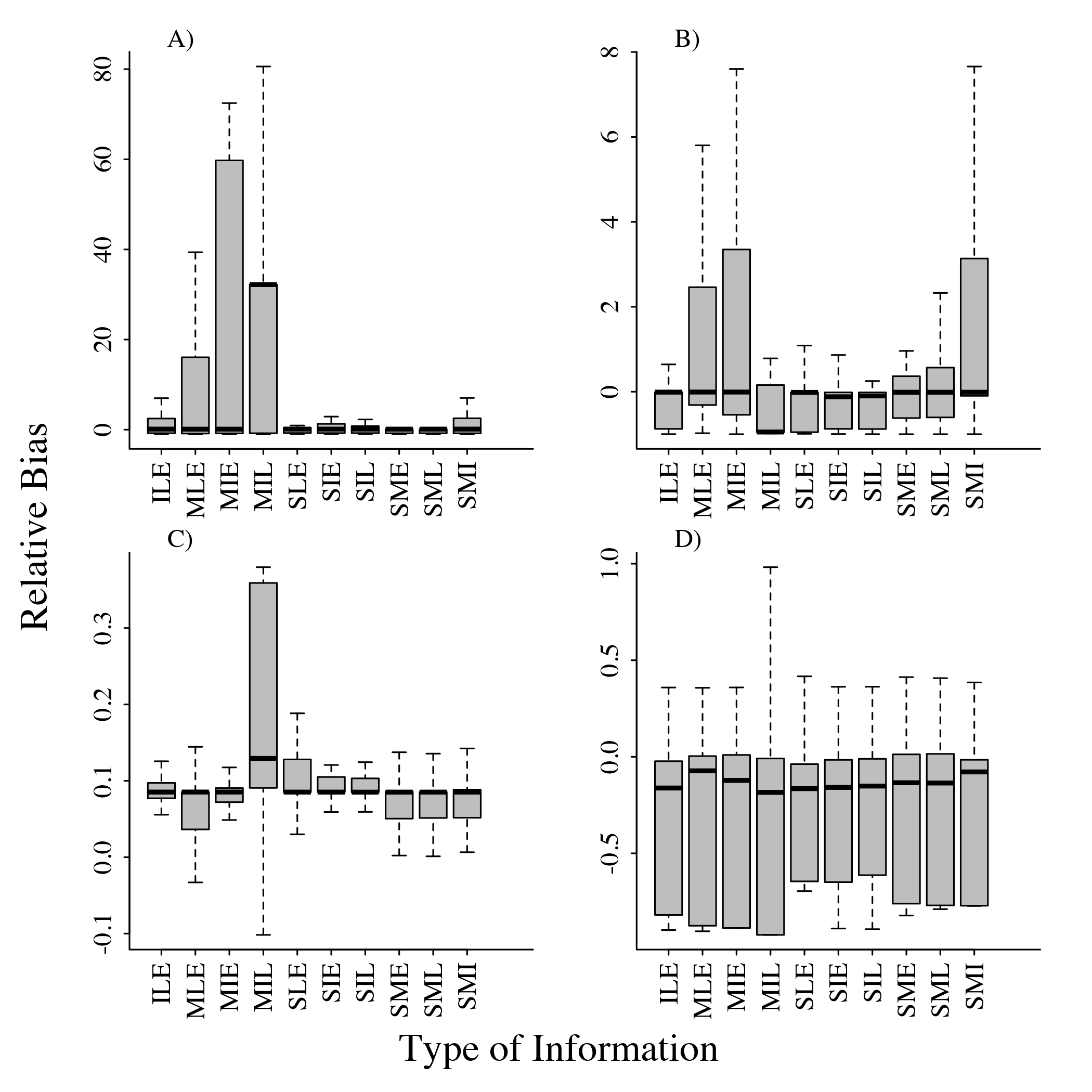

Supplement: S3 Fig — Labels in the x axis refer to the time series used for estimation. A) τ, B) θ, C) b0, D) b1. SMILE: Susceptible, Immune, Infected, LIZ, Environment. (TIFF) [file pone.0208621.s003.tiff]
