# Supplementary material for: Decoupling environmental effects and host population dynamics for anthrax, a classic reservoir-driven disease
Source: PLoS One. 2018 Dec 12;13(12):e0208621. doi: 10.1371/journal.pone.0208621 (PMC6291251; doi:10.1371/journal.pone.0208621)
Supplement: S1 Appendix — (PDF) [file pone.0208621.s004.pdf]

Decoupling environmental effects and bison (*Bison bison bison*) population dynamics in anthrax, a classic reservoir-driven disease

Juan Pablo Gomez, Dawn Nekorchuk, Liang Mao, Sadie J. Ryan, José Miguel Ponciano, Jason K. Blackburn.

S1 Appendix

Demonstration of the derivation of infection probability as a stochastic process with heterogeneity in dispersion effort

In what follows we derive the infection probability by considering heterogeneity in dispersion effort using three different assumptions. First, we assume that the probability of a new case does not depend on the total number of past infections, second we assume that the larger the number of past infections, the smaller the number of future infections that will be produced. The third case makes the opposite assumption of the second case, that is, the probability of a new infection increases with the number of past successful transmission events.

For the case in which the number of future infections is not dependent on previous infections but is simply a constant $\delta(x) = b$, the stochastic process modeling the accumulation of successful transmission encounters is given by a Poisson process [1]. We then introduce heterogeneity in the dispersion effort as a Gamma distributed random variable and integrate the Poisson probabilities over such distribution, as in Dennis [1]:

$$P(X(a) = x) = \int_0^\infty \frac{e^{-abE} abE^x}{x!} \frac{\theta^\tau}{\Gamma(\tau)} a^{\tau-1} e^{-\theta a} da$$

$$= \frac{(bE)^x \theta^\tau}{x!\Gamma(\tau)} \int_0^\infty e^{-a(bE+\theta)} a^{x+\tau-1} da$$

$$= \frac{(bE)^x \theta^\tau}{x!\Gamma(\tau)} \Gamma(x+\tau) \left(\frac{1}{bE+\theta}\right)^{x+\tau}$$

$$= \frac{\Gamma(x+\tau)}{x!\Gamma(\tau)} \left(\frac{bE}{bE+\theta}\right)^x \left(\frac{\theta}{bE+\theta}\right)^\tau$$

$$= \binom{x+\tau-1}{x} \left(\frac{\theta}{bE+\theta}\right)^\tau \left(\frac{bE}{bE+\theta}\right)^x$$

Thus, the process becomes a negative binomial process in which the variance is larger than the mean, and this allows for over-dispersion in the dispersion effort of the infectious agent. From this derivation, the probability of no infections is simply given by

$$P(X(a) = 0) = \left(\frac{\theta}{bE+\theta}\right)^\tau,$$

and $\lambda(t)$, the probability of one or more infections is then

$$\lambda(t) = P(x(a) \geq 1) = 1 - \left(\frac{\theta}{bE+\theta}\right)^{\tau}$$

$$= \frac{(bE+\theta)^{\tau} - \theta^{\tau}}{(bE+\theta)^{\tau}}.$$

Note that the time dependency in this expression is implicit in the E term (see equation 1 in the main text).

In the second case above, when the number of future infections is negatively influenced by the number of cases that the Local infectious zone has generated in the past we simply assume that $\delta(x) = b - cx$. Then, the number of successful transmission encounters is binomially distributed [1], and integrating over the gamma distribution modeling heterogeneity in the dispersion effort we get:

$$P(X(a) = x) = \int_0^{\infty} \binom{\frac{b}{c}}{x} \left(1 - e^{-acE}\right)^x \left(e^{-acE}\right)^{\frac{b}{c}-x} \frac{\theta^{\tau}}{\Gamma(\tau)} a^{\tau-1} e^{-\theta a} da$$

$$= \frac{\Gamma(y+1)\theta^{\tau}}{\Gamma(x+1)\Gamma(y-x+1)\Gamma(\tau)} \int_0^{\infty} \left(1 - e^{-acE}\right)^x \left(e^{-acE(y-x)}\right) a^{\tau-1} e^{-\theta a} da$$

$$= \frac{\Gamma(y+1)\theta^{\tau}}{\Gamma(x+1)\Gamma(y-x+1)\Gamma(\tau)} \int_0^{\infty} \left(1 - e^{-acE}\right)^x \left(e^{-a(cE(y-x)+\theta)}\right) a^{\tau-1} da,$$

where $y = \frac{b}{c}$. Knowing that $(1-x)^n = \sum_{k=0}^{n} \binom{n}{k}(-x)^k$, we can substitute $\left(1 - e^{-acE}\right)^x$ by $\sum_{k=0}^{x} \binom{x}{k} \left(-e^{-acE}\right)^k$, such that the RHS of the last equation becomes

$$= \frac{\Gamma(y+1)\theta^{\tau}}{\Gamma(x+1)\Gamma(y-x+1)\Gamma(\tau)} \int_0^{\infty} \sum_{k=0}^{x} \binom{x}{k} \left(-e^{-acE}\right)^k \left(e^{-a(cE(y-x)+\theta)}\right) a^{\tau-1} da$$

$$= \frac{\Gamma(y+1)\theta^{\tau}}{\Gamma(x+1)\Gamma(y-x+1)\Gamma(\tau)} \sum_{k=0}^{x} \int_0^{\infty} \binom{x}{k} (-1)^k \left(e^{-a(cEk+cE(y-x)+\theta)}\right) a^{\tau-1} da$$

$$= \frac{\Gamma(y+1)\theta^{\tau}}{\Gamma(x+1)\Gamma(y-x+1)\Gamma(\tau)} \sum_{k=0}^{x} \frac{(-1)^k \Gamma(x+1)}{\Gamma(k+1)\Gamma(x-k+1)} \int_0^{\infty} \left(e^{-a(cEk+cE(y-x)+\theta)}\right) a^{\tau-1} da$$

Now, since $\int_0^{\infty} x^{k-1} e^{-\frac{x}{\beta}} dx = \Gamma(k)\beta^k$ we can solve the integral in the RHS of the above equation:

$$= \frac{\Gamma(y+1)\theta^{\tau}}{\Gamma(y-x+1)\Gamma(\tau)} \sum_{k=0}^{x} \frac{(-1)^k}{\Gamma(k+1)\Gamma(x-k+1)} \Gamma(\tau) \left(\frac{1}{cE(k+(y-x)+\theta)}\right)^{\tau}$$

and just as before we can write explicitly the probability of no infection and the probability $\lambda(t)$ of one

or more infections:

$$P(X(a)=0)=\left(\frac{\theta}{cEy+\theta}\right)^{\tau} \quad \text{and hence}$$

$$P(X(a)\geq 1)=1-\left(\frac{\theta}{cEy+\theta}\right)^{\tau}$$

$$=1-\left(\frac{\theta}{cE\frac{b}{c}+\theta}\right)^{\tau}$$

$$=\frac{(bE+\theta)^{\tau}-\theta^{\tau}}{(bE+\theta)^{\tau}}.$$

In the third case, in which the number of future infections is positively influenced by the number of cases that the Local Infectious Zone has generated in the past we assume that $\delta(x)=b+cx$. In such case, the number of successful transmission encounters follows a negative binomial distribution [1] and integrating such distribution over the distribution of the dispersion effort we get that

$$P(X(a)=x)=\int_0^{\infty}\binom{\frac{b}{c}+x-1}{x}\left(e^{-acE}\right)^{\frac{b}{c}}\left(1-e^{-acE}\right)^{x}\frac{\theta^{\tau}}{\Gamma(\tau)}a^{\tau-1}e^{-\theta a}da$$

$$=\int_0^{\infty}\binom{y+x-1}{x}\left(e^{-acEy}\right)\left(1-e^{-acE}\right)^{x}\frac{\theta^{\tau}}{\Gamma(\tau)}a^{\tau-1}e^{-\theta a}da$$

$$=\binom{y+x-1}{x}\frac{\theta^{\tau}}{\Gamma(\tau)}\int_0^{\infty}\left(e^{-acEy}\right)\left(1-e^{-acE}\right)^{x}a^{\tau-1}e^{-\theta a}da$$

where again we set $y=\frac{b}{c}$. Proceeding as before, we substitute $\left(1-e^{-acE}\right)^{x}$ by $\sum_{k=0}^{x}\binom{x}{k}\left(-e^{-acE}\right)^{k}$, such that the RHS of the above equation becomes

$$=\binom{y+x-1}{x}\frac{\theta^{\tau}}{\Gamma(\tau)}\sum_{k=0}^{x}\binom{x}{k}(-1)^{k}\int_0^{\infty}\left(e^{-a(cEk+cEy+\theta)}\right)a^{\tau-1}e^{-\theta a}da$$

$$=\binom{y+x-1}{x}\frac{\theta^{\tau}}{\Gamma(\tau)}\sum_{k=0}^{x}\binom{x}{k}(-1)^{k}\Gamma(\tau)\left(\frac{1}{cEk+cEy+\theta}\right)^{\tau}$$

$$=\binom{y+x-1}{x}\theta^{\tau}\sum_{k=0}^{x}\binom{x}{k}(-1)^{k}\left(\frac{1}{cEk+cEy+\theta}\right)^{\tau}$$

And here again, we get that

$$P(X(a)=0)=\left(\frac{\theta}{cEy+\theta}\right)^{\tau} \quad \text{so that}$$

$$\lambda(t)=P(X(a)\geq 1)=1-\left(\frac{\theta}{cEy+\theta}\right)^{\tau}$$

$$=1-\left(\frac{\theta}{cE\frac{b}{c}+\theta}\right)^{\tau}$$

$$=\frac{(bE+\theta)^{\tau}-\theta^{\tau}}{(bE+\theta)^{\tau}}.$$

Derivation of R_0 for cases 2 and 3

Noting that in the case where the process $X(a)$ counts the number of successful transmissions of a single carcass introduced in a population of non-infected individuals, then its expected value, $E[X(a)]$ is in fact equal to the mean number of secondary infections R_0 in the context of this disease transmission setting. Using the closed form expressions for $P(X(a)=x)$ derived above for each one the three cases ($\delta(x)=b-cx$, $\delta(x)=b+cx$ and $\delta(x)=b$), we can easily derive analytical expressions for such expected value. The simplest case, when $\delta(x)=b$ is the case treated in the main text. Below we provide expressions for the two other cases.

First, we treat the case where $\delta(x)=b-cx$. Since $X(a)|a\sim \mathrm{Bin}(\mathrm{y},\mathrm{p}=1-\mathrm{e}^{-\mathrm{acE}})$, then $E[X(a)|a]=yp=y\left(1-e^{acE}\right)=y-ye^{-acE}$, so,

$$\mathrm{E[X]}=\int_0^{\infty}\left(y-ye^{-acE}\right)\frac{\theta^{\tau}}{\Gamma(\tau)}a^{\tau-1}e^{-\theta a}da$$

$$=y-y\int_0^{\infty}\frac{\theta^{\tau}}{\Gamma(\tau)}a^{\tau-1}e^{-a(\theta+cE)}da$$

$$=y-y\frac{\theta^{\tau}}{(\theta+cE)^{\tau}}\int_0^{\infty}\frac{(\theta+cE)^{\tau}}{\Gamma(\tau)}a^{\tau-1}e^{-a(\theta+cE)}da$$

Because the integral above is 1 it follows that,

$$\mathrm{E[x]}=\mathrm{R}_0=\mathrm{y}\left(1-\frac{\theta^{\tau}}{(\theta+\mathrm{cE})^{\tau}}\right)=\frac{\mathrm{b}}{\mathrm{c}}\left(1-\frac{\theta^{\tau}}{(\theta+\mathrm{cE})^{\tau}}\right)$$

For the other case where $\delta(x)=b+cx$ we get that

$$\mathrm{E[X(a)|a]}=\frac{\frac{b}{c}\left(1-e^{-acE}\right)}{e^{-acE}}$$

$$=\frac{b}{c}\left(1-e^{-acE}\right)e^{acE}$$

$$=\frac{b}{c}e^{acE}-\frac{b}{c}\left(e^{-acE}\right)e^{acE}.$$

Simplifying and integrating we get

$$\mathrm{E[X]} = \frac{b}{c}\int_0^\infty e^{acE}\frac{\theta^\tau}{\Gamma(\tau)}a^{\tau-1}e^{-\theta a}\partial a - \frac{b}{c}$$

$$= \frac{b}{c}\frac{\theta^\tau}{(\theta - cE)^\tau}\int_0^\infty \frac{(\theta - cE)^\tau}{\Gamma(\tau)}a^{\tau-1}e^{-a(\theta-cE)}\partial a - \frac{b}{c}$$

$$= \frac{b}{c}\frac{\theta^\tau}{(\theta - cE)^\tau} - \frac{b}{c}$$

Thus,

$$\mathrm{E[X]} = \mathrm{R}_0 = \frac{\mathrm{b}}{\mathrm{c}}\left(\frac{\theta^\tau}{(\theta - \mathrm{cE})^\tau} - 1\right).$$

References

1. Dennis B. Allee effects: population growth, critical density, and the chance of extinction. Natural Resource Modeling. 1989;3(4):481–538.
