# Supplementary material for: Decoupling environmental effects and host population dynamics for anthrax, a classic reservoir-driven disease
Source: PLoS One. 2018 Dec 12;13(12):e0208621. doi: 10.1371/journal.pone.0208621 (PMC6291251; doi:10.1371/journal.pone.0208621)
Supplement: S2 Appendix — Data is presented on a daily basis and only for the range of weeks for which there was at least one anthrax case reported. Date is in Month/Day/Year format. (PDF) [file pone.0208621.s005.pdf]

Decoupling environmental effects and bison (*Bison bison bison*) population dynamics in anthrax, a classic reservoir-driven disease

Juan Pablo Gomez, Dawn Nekorchuk, Liang Mao, Sadie J. Ryan, José Miguel Ponciano, Jason K. Blackburn.

S2 Appendix

Observed number of cases during the 2008 Montana anthrax outbreak. Data is presented on a daily basis and only for the range of weeks for which there was at least one anthrax case reported. Date is in Month/Day/Year format.

Date	Week	Deaths
7/23/08	30	2
7/31/08	31	5
8/1/08	31	19
8/2/08	31	11
8/3/08	32	18
8/4/08	32	28
8/5/08	32	27
8/6/08	32	56
8/7/08	32	12
8/8/08	32	6
8/9/08	32	18
8/10/08	33	13
8/11/08	33	7
8/12/08	33	5
8/13/08	33	4
8/14/08	33	2
8/15/08	33	3
8/16/08	33	3
8/17/08	34	11
8/18/08	34	5
8/19/08	34	1
8/20/08	34	4
8/21/08	34	1
8/22/08	34	4
8/23/08	34	6
8/24/08	35	7
8/25/08	35	0
8/26/08	35	0
8/27/08	35	0
8/28/08	35	0
8/29/08	35	2
8/30/08	35	0
8/31/08	36	0
9/1/08	36	0
9/2/08	36	0
9/3/08	36	4
